# Physical activity and body composition outcomes of the GreatFun2Run intervention at 20 month follow-up

**DOI:** 10.1186/1479-5868-8-74

**Published:** 2011-07-18

**Authors:** Trish Gorely, John G Morris, Hayley Musson, Susie Brown, Alan Nevill, Mary E Nevill

**Affiliations:** 1 Institute of Youth Sport, School of Sport and Exercise Sciences, Loughborough University, Loughborough, LE11 3TU, UK; 2 University of Wolverhampton, School of Sport, Performing Arts and Leisure, Walsall Campus, Gorway Road, Walsall, WS1 3BD, UK

**Keywords:** Physical activity, intervention, children, long term follow-up

## Abstract

**Background:**

Physical inactivity is recognised as a public health concern within children and interventions to increase physical activity are needed. GreatFun2Run was a school-based healthy lifestyles intervention that showed positive changes in physical activity levels and body composition immediately post-intervention. The purpose of this paper was to examine whether these changes in physical activity and body composition were maintained 18-20 months after the intervention ended.

**Method:**

Participants (n = 589, aged 7-11 yrs) from 4 intervention and 4 control schools took part in the 10-month intervention, of which 421 (71%) were present for follow-up. The intervention comprised a CD-rom learning and teaching resource for teachers; an interactive website for pupils, teachers and parents; two highlight physical activity events (1 mile school runs/walks); a local media campaign; and a summer activity wall planner and record. Randomisation was not possible because of local media content. Outcome measures were objectively measured physical activity (pedometers and accelerometers) and body composition variables (body mass index, waist circumference, estimated percent body fat, and sum of skinfolds). Teacher interviews and participant focus groups were conducted. Multi-level modelling was employed for the data analysis.

**Results:**

Both control and intervention participants had increased their physical activity at follow-up but there was no group by time interaction (control: 2726 steps per day increase; intervention 3404 steps per day increase, p > .05). There were significant increases in estimated percent body fat, sum of skinfolds, waist circumference and body mass index (BMI) with increasing age. In the control group, there was evidence for a plateauing in the rate of change in all body composition variables with increasing age, except BMI. In contrast, significant interaction terms suggest that the rate of change in waist circumference, BMI and BMISDS continued to increase with age in the intervention group. Teacher interviews suggested that because of time pressures, competing resources, curriculum demands and staff changes the majority of teachers had not continued to use the resources.

**Conclusions:**

While the intervention initially produced positive changes in physical activity levels and body composition, these changes were not sustained once the intervention ended. Facilitating long-term health behaviour change in children remains a challenge.

## Background

The importance of regular physical activity for healthy growth and development in children has been widely recognised [1,2]. However, a significant number of young people fail to meet current physical activity guidelines of 60 minutes of physical activity on most days of the week [2]. As a consequence there is a need for effective interventions to encourage long-term participation in healthy lifestyles in young people [3].

Two extended school-based interventions have demonstrated that it is possible to facilitate long-term health behaviour change in primary school aged children [4,5]. Manios et al reported on a primary school-based intervention involving changes to the physical education curriculum plus annual workbooks covering dietary issues, physical activity and fitness and other health behaviours. At the end of the 6 year intervention changes in physical activity and dietary changes significantly favoured the intervention group [6]. Four years after the intervention ended physical activity levels had declined in both intervention and control participants, but remained significantly higher in intervention males but not females [4]. Likewise, the school and family based CATCH programme, conducted over 3 school years when participants were in grades 3 through 5 (ages ~8-10 years), showed significant post-intervention effects for vigorous physical activity and daily intakes of energy from total fat and saturated fat. At three year follow-up, when participants were in grade 8 (age ~13 years), significant differences favouring the intervention participants remained for both diet and physical activity variables, although the size of the differences had attenuated [5]. Both these studies took a similar intervention approach, delivering non-competitive forms of exercise during physical education classes, delivering classroom based health lessons, and encouraging parental involvement. Although both these studies had positive short- and long-term intervention effects because of cultural and educational differences between countries questions have been raised about the appropriateness of taking interventions from one country and implementing them in another [7,8].

Within the UK itself there is limited evidence from primary school based interventions, with only two randomised controlled trials identified [9-11], both of which only report on post-intervention results. The lack of long-term follow-up results is reflective of a limitation in the wider field. For example, a recent series of reviews for the National Institute for Health and Clinical Excellence UK [12], showed that while there are a number of interventions aimed at increasing physical activity in young people, few of these (20%) had follow-up periods greater than 6 months, and the majority had no follow-up period (67%). Other reviews have also highlighted the need for longer follow-up periods [7,13-15], as without sustained follow-up periods (in the order of 1-2 years) the maintenance of any intervention effects cannot be assessed [13,14].

GreatFun2Run was a 10-month primary school-based intervention designed for use with 7-11 year old children. The post-intervention results of GreatFun2Run [16] showed a significant effect on physical activity. Specifically relative to children in control schools, those in intervention schools significantly increased their daily steps (3059 steps per day increase vs. 1527 steps per day increase), total time in moderate-to-vigorous physical activity (MVPA) (by 9 minutes/day vs. a decrease of 10 minutes/day), and their time in MVPA bouts lasting at least one minute (10 minutes/day increase vs. no change). Additionally, older participants in intervention schools showed a significant slowing in the rate of increase in estimated percent body fat (intervention 0.9% vs. control 1.8% per year of age), BMI (intervention 0.4 vs. control 0.9 BMI units per year of age), BMI-SDS (intervention -.05 vs. control 0.12 per year of age), and waist circumference (intervention 1.8 cm vs. control 2.8 cm per year of age). However, there were no differences between groups in fruit and vegetable consumption, aerobic fitness, knowledge of healthy lifestyles, perceived competence, enjoyment of physical activity, or intrinsic motivation. Extrinsic motivation decreased significantly more in the intervention group. The purpose of this paper was to examine whether the significant changes in physical activity and body composition post intervention were maintained approximately 18-20 months after the intervention ended.

## Method

### Participants

Four primary schools in the north-east of England who had already agreed to take part in the "GreatFun2Run" programme were recruited for this study (540 schools in total participated in the programme). These schools were matched with 4 schools in the East Midlands of England on the basis of size, ethnicity and socioeconomic status, as reflected in the Index of Multiple Deprivation (IMD) for the school postcode. The IMD is a measure of compound social and material deprivation, calculated from a variety of data including income, employment, health, education, and housing. All participating primary schools were government-funded schools.

In total 589 children (310 intervention, 279 control; 287 boys, 302 girls) took part in the evaluation, of which 421 (71%) were present for follow-up. The mean age of children at baseline was 8.8 years in the intervention schools and 8.9 years in the control schools. The majority of participants were of white British ethnicity (intervention 94.8%, control 96.5%). Despite matching schools as closely as possible on the IMD associated with the school postcode (as a broad reflection of the school catchment area) there were differences in socioeconomic status at the individual level between the two groups, with the intervention group being of lower socio-economic status than the control group when measured by the IMD for the postcode defined ward in which each participant resided. These differences were paralleled in household income with income in intervention schools being significantly lower (it is worth noting though that over 50% of parents chose not to supply this information).

**Figure 1:**
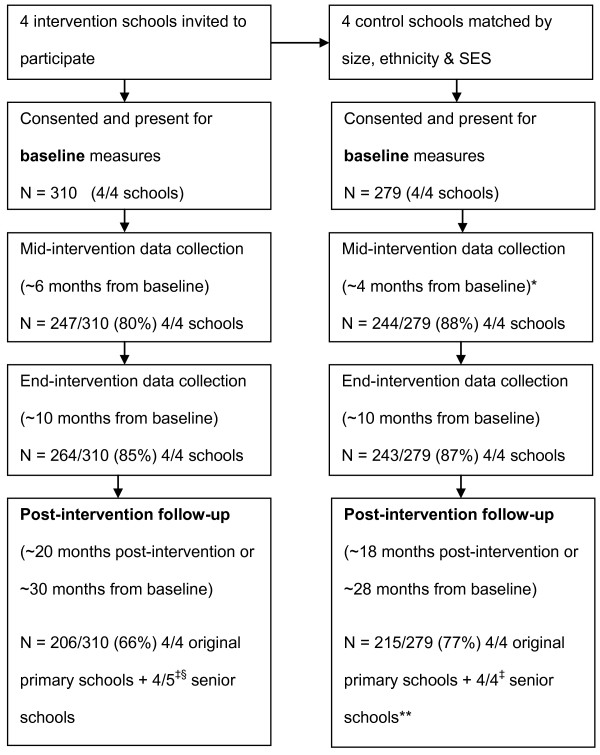
**Flow of schools and participants through study**. * difference in time to follow-up 1 between control and intervention schools was the result of the scheduling of the Christmas school holidays which meant the first data collection could not occur. These time differences are accounted for with the analysis procedures undertaken. ‡ pupils in year 5 at baseline had moved on to senior schools at post-intervention follow-up. § one senior school would not allow access (n = 30).

The flow of schools and participants through the project is depicted in Figure 1. Approximately a third of participants (34.8%) had finished primary school and moved on to secondary schools at follow-up. One secondary school attended by intervention school pupils declined access to their pupils accounting for 30 of those not available for follow-up. This refusal was reflected in a greater than expected absence at follow-up of pupils from intervention schools relative to control schools (Chi^2^(1) = 8.11, p <.05), and given the lower SES of participants from intervention schools at baseline, a greater number of those absent at follow-up were of lower SES (Chi^2^(2) = 12.2, p <.05). However, there was no difference (p > .05) at baseline in age, body composition, steps/day, or minutes of MVPA between those present at follow-up and those absent. In addition, there were no differences in the proportions of boys and girls present at follow-up compared to baseline (p > .05).

The study was approved by the Ethical Advisory Committee of Loughborough University and the head teachers of participating schools. Parental consent was obtained prior to each round of data collection and parents also completed a health screening questionnaire on behalf of their child. On the day of testing all participants indicated their assent to participate and were asked to indicate that they were free of illness. A small number of children were excluded from the Multi-stage Shuttle Running Test for medical reasons (e.g., uncontrolled asthma, family history of early coronary death).

### Intervention

The intervention has been described in detail in Gorely et al [16] and only a brief overview is provided here. The GreatFun2Run intervention was designed and implemented by Great Run (a sports marketing and event management company). The programme aimed to increase children's activity levels through PE lessons that taught the skills of running (via a number of sports and activities), through highlight running/walking events which gave a goal to work towards, and through a range of classroom activities that reinforced children's learning and encouraged them to reflect on their activity levels and to do more voluntarily. Healthy food choices were explained and encouraged in a holistic approach to children's health education. The programme was multifaceted and comprised:

i. a CD-rom learning and teaching resource for teachers with physical education lesson plans and homework exercises plus suggestions for including health and activity related issues across the curriculum in literacy, numeracy, history, design, science, and geography lessons. The CD-rom was themed around space travel and contained 8 planets (units of work) the teachers could visit and work through, covering topics including healthy eating, self-evaluation of physical levels, and how our bodies work. The CD-rom also introduced the "10 Star Rules" for good nutrition and physical activity which underpinned the programme;

ii. two highlight events (1 mile run/walks) to give the children a goal for increasing their physical activity. These events were mass participation events with the emphasis on participation not competition;

iii. an interactive website for pupils, teachers and parents to raise awareness of the need for physical activity and healthy eating. This website supported and expanded on the key health and fitness messages from the CD-rom;

iv. a local media campaign employing regional radio and print media to maintain interest and create excitement;

v. a summer activity wall planner and record.

The programme was designed to be as flexible as possible and teachers could decide when and how they used the material provided. No specific training was provided for the teachers and all instructions were contained within the pack. Parents were engaged through homework tasks, information and publicity relating to runs, the activity planner, and by access to the web site. The control schools continued with their usual physical education and health curriculum.

### Measures

#### Physical activity

Daily physical activity was assessed objectively in 2 ways. All participants wore a Digiwalker SW200 pedometer for one week during waking hours. Children recorded the total number of steps taken in the previous 24 hours at the start of each school day. The steps recorded on Monday morning related to the previous 3 days and participants indicated whether they had worn the pedometer for most of Saturday and Sunday. In addition to the pedometer approximately 50% of children also wore an ActiGraph GT1M accelerometer during waking hours for this week. The sampling epoch was 5 seconds. During data processing 20 minutes of consecutive zero's was considered indicative of non-wearing and these data were deleted, minimum day length was set at 9 hours and time spent in moderate to vigorous physical activity (MVPA) was calculated using the Freedson et al [17] age specific cutpoints. Accelerometer data is reported in two ways: (i) total time in MVPA regardless of bout length (MVPA_total_) and (ii) total time in MVPA when only bouts of at least 1 minute duration were included (MVPA_bout_). When defining a bout an interruption of no more than 10% of epochs was allowed (i.e. within any given bout individuals could drop below the MVPA cut-off for no more than 10% of the time). For both pedometers and accelerometers the first day of recording was dropped to account for likely reactivity and a minimum of 3 weekdays and 1 weekend day was required for inclusion of a participant's data in the study results.

#### Anthropometric measures

Height was measured (to the nearest 0.1 cm) using a stadiometer (Leicester Height Measure, seca ltd., England). Body mass was measured (to the nearest 0.01 kg) using portable digital scales (seca 770, seca ltd, Birmingham, UK). This was used to calculate body mass index (BMI) which was subsequently converted to age and gender specific standardised scores using the 1990 growth curves from Cole et al., [18]. Subscapular and triceps skinfold thickness was assessed using callipers (Harpenden, Baty International, England) and were taken by two trained research assistants following standardised Level 1 International Society for the Advancement of Kinanthropometry protocols. Body fat percentage was then estimated using generalised equations for prepubescent boys and girls [19]. Sum of skinfolds was calculated by adding together the subscapular and triceps skinfolds scores. Waist circumference was measured (to the nearest 0.1 cm) at the widest part of the torso between the xiphoid process of the sternum and the iliac.

#### Procedures

A field team of 10 researchers visited each school for a day 4 times during the evaluation period (baseline, midway (4-6 months post-baseline), end of intervention (~10 months post-baseline), and follow-up (28-30 months post-baseline). All measures were completed at all testing points. Participants attended two sessions of 90 minutes each on each testing day in groups of 15-30. In one session they completed the anthropometric assessments and the multi-stage shuttle test. In the other session they completed the psychological measures, the knowledge test, and the food recall interview. They were also given pedometers and, in 50% of the sample, accelerometers. Participants were also instructed on how and when to wear these devices during this session. Due to the differences in geographical location it was not possible to blind the measurement team to the intervention and control group allocation. A week after the testing a researcher returned to the school to collect the pedometers and accelerometers. This paper reports on only physical activity and body composition results.

At the final data collection a sub-sample of pupils from each class were invited to take part in a focus group to explore their recollection and experience of GF2R. Eleven focus groups (n = 72, 34 boys, group size 4-8 participants) were conducted and followed a semi-structured interview schedule. Each focus group lasted between 15 and 20 minutes. In addition, eight teachers who had been involved in the GF2R programme were interviewed using a semi-structured schedule examining recall of GF2R, opinions of the programme and its impact, and why they had or had not continued to use the resources. The focus groups and interviews were recorded using an audio voice recorder and were then transcribed verbatim.

#### Analysis of data

We applied a multilevel statistical model using ML-win [20] to assess changes in physical activity and body composition. Multilevel modelling is an extension of ordinary multiple regression, where the data have a hierarchical or clustered structure. A hierarchy consists of units or measurements grouped at different levels. Initially a 3 level hierarchy was explored, based on the idea that individuals within a class are more like each other than individuals between classes, and individuals within a school are more like each other than individuals between schools. However, the variances associated with both school and class were not significant and we could not justify their inclusion as levels within the model. A two level repeated measures model, with individuals at *level 2 *and the participants' repeated measurements at *level 1 *produced models with the best fit statistics. Time in study was measured in months, so that the differences in time periods between testing for different participants could be accounted for. The regression coefficients for time in intervention therefore reflect a change per month. The change across the whole intervention period can be estimated by multiplying this value by total months in the study (in examples, 29 months has been used as this is the average follow-up period). To account for potential school effects dummy variables were created for each school and included as a fixed factor within the model. Backward elimination was employed to remove non-significant schools, and only schools that improved the fit of the model were included. The potential confounders of age, gender and socio-economic status were entered into all models, but in the interests of parsimony were only retained if their inclusion resulted in a significant improvement in fit statistics.

Participants were included in the analysis regardless of how many testing sessions they actually attended. All analyses were conducted on an intention-to-treat basis. Figure 1 shows the number of participants tested at each session. As the purpose of this paper was to evaluate the long-term effects of the intervention only data from the baseline and follow-up data collections are included in the current analysis. The results immediately post-intervention using the mid-intervention and post-intervention data points are published in Gorely et al [16].

Within the results tables the β coefficients represent the difference in the dependent variable by the units of the fixed parameter. The reference category for gender is boys and the control group is the reference category for group. For example, for the steps outcome the β coefficient for gender represents the difference in steps for girls relative to boys, the β coefficient for time in study represents the average change in steps for each month of the study, and the β coefficient for the time by group interaction represents the difference in change in steps for the intervention group relative to the control group. 95% confidence intervals were used to indicate whether a difference is significant or not. If the confidence interval contains zero then the associated parameter estimate is not significant.

Transcripts of the teacher interviews and pupil focus groups were analysed by question area to identify key themes.

## Results

Table 1 presents the absolute values at baseline and follow-up for physical activity and body composition variables.

**Table 1:** Absolute values (mean, (SD)) at baseline and follow-up for physical activity and body composition variables

Variable	Intervention			Control		
	**Boys**	**Girls**	**Overall**	**Boys**	**Girls**	**Overall**

Steps per day						
Baseline	9789.3 (2929.1)	9397.8 (2559.4)	9579.4 (2735.6)	11178.0 (3662.5)	9452.4 (2654.7)	10278.5 (3284.3)
Follow-up	15007.7 (4250.7)	13393.3 (3573.0)	14213.3 (3998.9)	14663.5 (4182.6)	12978.8 (3507.6)	13775.0 (3919.8)
MVPA total						
Baseline	138.9 (26.4)	113.9 (21.5)	124.7 (26.7)	125.4 (26.1)	116.6 (21.1)	120.3 (23.7)
Follow-up	141.3 (36.0)	119.0 (33.1)	128.3 (35.7)	125.5 (29.1)	104.6 (30.0))	114.1 (31.1)
MVPA bouts						
Baseline	52.3 (18.7)	30.7 (12.4)	40.1 (18.7)	44.7 (18.4)	30.4 (11.5)	36.5 (16.4)
Follow-up	65.2 (27.0)	45.3 (27.6)	53.7 (28.8)	57.4 (21.9)	36.7 (20.0)	46.1 (23.2)
Estimated body fat percentage						
Baseline	18.5 (6.4)	26.7 (5.6)	22.6 (7.2)	17.6 (6.6)	25.8 (5.6)	21.7 (7.4)
Follow-up	21.4 (9.4)	28.0 (6.7)	24.7 (8.8)	20.2 (8.3)	26.7 (6.0)	23.6 (7.8)
Sum of skinfolds						
Baseline	20.0 (8.5)	25.9 (11.2)	23.0 (10.3)	19.1 (8.9)	23.8 (10.3)	21.5 (9.9)
Follow-up	23.9 (13.1)	28.3 (13.3)	26.1 (13.3)	22.5 (11.5)	26.1 (11.9)	24.4 (11.8)
Waist circumference						
Baseline	60.5 (6.9)	60.1 (8.9)	60.3 (7.9)	60.8 (7.5)	58.5 (6.7)	59.6 (7.2)
Follow-up	65.7 (7.7)	65.0 (9.8)	65.4 (8.8)	66.0 (7.4)	63.7 (7.3)	64.8 (7.4)
BMI						
Baseline	17.7 (2.7)	18.1 (3.1)	17.9 (2.9)	17.2 (2.4)	17.5 (2.6)	17.3 (2.5)
Follow-up	18.9 (3.3)	19.6 (3.8)	19.3 (3.6)	18.8 (3.0)	18.8 (2.9)	18.8 (2.9)
BMI-SDS						
Baseline	0.6 (1.1)	0.6 (1.1)	0.6 (1.1)	0.4 (1.1)	0.4 (1.0)	0.4 (1.1)
Follow-up	0.6 (1.2)	0.5 (1.2)	0.6 (1.2)	0.5 (1.2)	0.3 (1.0)	0.4 (1.1)

### Physical Activity

Table 2 shows the results of the multi-level regression analysis for steps per day, MVPA_total_, and MVPA_bout_. From baseline to follow-up all participants significantly increased their daily steps (controls: ~94 steps per study month; intervention by ~117 per study month), equating to around 2726 steps per day more in controls and 3404 steps more per day in the intervention group. This group by time difference was not significant. A similar pattern was observed for MVPA_bout _with all participants increasing their time in sustained bouts of MVPA (controls: ~ 0.3 minutes for every study month; intervention: ~ 0.4 minutes for every study month) equating to around 9 minutes more per in the control group and 12 minutes in the intervention group. But again this group by time difference was not significant. At follow-up there was small, but not significant, change in control participants daily time in MVPA_total _(~0.2 minutes for every study month, equating to around 6 minutes more per day at follow-up). Intervention participants showed a slightly larger increase in MVPA_total _(~0.6 minutes for every study month, equating to around 17 minutes more per day at follow-up). However, this group by time difference was not significant. For all physical activity measures the within pupils between time variance was larger than the between pupil variances.

**Table 2:** Multilevel regression analysis for steps/day, and minutes of MVPA

	**Steps/day**^**± **^		**MVPA**_**total **_**(mins/day) **^**± **^		**MVPA**_**bout **_**(mins/day) **^**± **^	
	**β**	CI*	**β**	**CI**	**β**	**CI**

Constant	11281.5^†^	10641.3, 11921.6	126.2^†^	119.2, 133.2	44.9^†^	40.0, 49.8
Gender^¥^	-1260.6^†^	-1824.2, -696.9	-18.0^†^	-24.5, -11.5	-18.0^†^	-22.5, -13.6
Group**	-1434.6^†^	-2223.6, -645.6	3.3	-4.1, 10.7	5.4	-0.1, 11.0
Time in study (months)	93.8^†^	59.3, 128.4	.23	-0.2, 0.6	0.3^†^	0.1, 0.5
Group × time in study (month)	23.6	-8.5, 55.7	.35	-0.1,0.7	0.1	-0.1, 0.4
Age	377.5^†^	52.6, 702.4	-5.6^†^	-9.2, -1.9	n/a	
School 1	1172.9^†^	1093.5, 1252.2	n/a		n/a	
School 2	1996.4^†^	1014.8, 2977.9	n/a		n/a	
School 6	n/a		n/a		7.181^†^	1.3, 13.1

Random parts	variance	CI	variance	CI	variance	CI

Level 2 (Between individuals)	3486392.8^†^	1942487, 5030299	282.7^†^	139.2, 426.3	89.9^†^	15.3, 164.4

Level 1 (Within individuals)	7545611.0^†^	6077926, 9013296	395.8^†^	273.1, 518.6	247.7^†^	172.8, 322.5

Notes:^± ^The inclusion of SES did not improve the fit of the model and it was therefore not included.

*CI = 95% confidence interval; with level 2 variation (Between individuals) and with level 1 variation (within individuals or repeated measures)

^† ^= significant at p < 0.05;^¥ ^Reference category = boys; ** reference category = control.

### Body Composition

Table 3 shows the results of the multi-level regression analysis for estimated body fat percentage, sum of skinfolds, waist circumference, BMI, and BMI-SDS. There were significant increases in estimated percent body fat, sum of skinfolds, waist circumference and BMI with increasing age. In the control group, significant negative coefficients for the age^2^ term provide evidence for a plateauing in the rate of change in all body composition variables, except BMI, with increasing age. The group by age^2^ interactions were significant for waist circumference, BMI and BMISDS and suggest that the rate of change in these variables continues to increase with age in the intervention group.

**Table 3:** Multilevel regression analysis for body composition variables

	**Estimated % body fat**^**± **^		**Sum of skinfolds**^**± **^		**Waist circumference**^**± **^	
	**β**	CI*	**β**	**CI**	**β**	**CI**

Constant	19.1^†^	18.1, 20.1	20.8^†^	19.2, 22.3	62.6^†^	61.5,63.7
Sex^¥^	7.5^†^	6.4, 8.5	5.0^†^	3.3, 6.6	-1.2^†^	-2.4,-0.03
Group**	1.3^†^	0.1, 2.5	0.4	-1.5, 2.3	0.9	-0.5, 2.2
Age	0.8^†^	0.7, 1.0	1.4^†^	1.2, 1.7	2.3^†^	2.1, 2.4
Age^2^	-0.2^†^	-0.3, -0.03	-0.3^†^	-0.6, -0.1	-0.2^†^	-0.3, -.01
Group × Age^2^	0.2	-0.03, 0.4	0.3	0.0, 0.7	0.4^†^	0.1, 0.6
School1	-1.8^†^	-3.4, -0.1	n/a		-2.0^†^	-3.8, -0.1
School4			3.5^†^	0.8, 6.3	n/a	

Random parts	variance	CI	variance	CI	variance	CI

Level 2 (Between individuals)	34.5^†^	2.5, 29.6	89.0^†^	6.4, 76.5	44.6^†^	3.1, 38.5

Level 1 (Within individuals)	10.0^†^	0.7, 8.6	25.5^†^	1.8, 21.9	11.2^†^	0.8, 9.7

	BMI^± ^		BMI-SDS^± ^			
	β	CI	β	CI		

Constant	17.8^†^	17.3, 18.2	0.5^†^	0.3, 0.6		
Sex^¥^	0.4	-0.05, 0.9	-0.1	-0.2, 0.1		
Group**	0.4	-0.03, 0.9	0.1	-0.04, 0.3		
Age	0.6^†^	0.6, 0.7	0.02	0.0, 0.04		
Age^2^	-0.04	-0.1, 0.01	-0.03^†^	-0.05, -0.01		
Group × Age^2^	0.1^†^	.05, 0.2	0.04^†^	0.02, 0.1		

Random parts	variance	CI	variance	CI		

Level 2 (Between individuals)	7.5^†^	0.5, 6.6	1.1^†^	0.1, 1.0		
Level 1 (Within individuals)	1.0^†^	0.1, 0.9	0.1^†^	0.01, 0.1		

Notes:^± ^The inclusion of SES did not improve the fit of the model and it was therefore not included.

*CI = 95% confidence interval; with level 2 variation (Between individuals) and with level 1 variation (within individuals or repeated measures)

^† ^= significant at p < 0.05;^¥ ^Reference category = boys; ** reference category = control.

### Feedback from teachers

The majority of teachers interviewed recalled the GreatFun2Run programme and that the resources had been useful for generating additional ideas for activities, but only 2 of the 8 teachers said they were currently using any of the resources. Both of these teachers commented particularly on the cross-curricula links. For example, one teacher said:

I have (used the resources), not all of the resources but I certainly have used the numeracy, they had graphs and that kind of thing, so I used the numeracy and there was some literacy material as well, talking about various things. And we used the science ones, in revision as the children approached their SATs *[Standardised Assessment Tests]*. I took them out of the pack and used them in my planning file and the children enjoyed them.

Reasons given for not continuing to use the resources varied but reflected time issues, competing resources, curriculum demands, and not teaching PE, as specialists were now coming into the school. For example one teacher commented, "to be honest we didn't use the resource pack for the planning base, we've got lots of different resources, but we went mainly from the objectives in the QCA *[Qualification and Curriculum Authority] *units". Similarly, another teacher explained: "We have used it, I have used it minimally I must admit, you've got to make time, and we did use bits and pieces that fitted in with the way that the curriculum is run in this school."

Factors perceived to influence the continued use and impact of the programme included staffing changes, school leadership, and continuing professional development (CPD) support. For example, a number of teachers felt that the impact of the programme was restricted due to a limited ability to follow through the intervention with the same pupils. As one teacher stated:

I think part of the problem has been obviously with people changing roles and that staff turnover has been high so there's been different people doing different jobs. I think as well if the same class teachers had been in the same year groups and followed those children through, then they'd have known what background had already been done and to build on it from there. I think that is a little bit of an issue. But yes, I think it's been positive for the children involved and its just, as I said, given them a fair insight into a healthy lifestyle for the future.

Teachers again stated the importance of strong leadership from the head teacher in sustaining the programme. They also felt that follow-up support would have been beneficial and would help the long-term sustainability of the programme. As explained by one teacher:

Whole school training, CPD, they've got to come in and do that, you get these resources, you hand them out but its having the time to actually, I mean I've spoken to all the members of staff about it, given them the resources, but the people who actually create it, if they came in and talked to the teachers that would really help.

The final factor influencing the impact of the programme that was raised by teachers was the importance of support from the family. As one teacher stated "I think a lot of it is home life, if the parents don't push them towards sporting activities then you're fighting a battle straight away in school".

### Feedback from pupils

Pupils recalled various elements of the programme (e.g., the run/walks, the testing, the wall planner and the website) and although not always recognising them as from the "10 star rules", they could recall many of the key messages (e.g., 5 fruit and vegetables a day, eat breakfast, drink water, 60 minutes PA a day etc). Most felt that the different components of the programme had helped them do more activity at the time and that the programme had encouraged them to try new activities. The vacation wall planner appeared to have been popular and had encouraged them to do activities during the holidays so that they could fill in the chart. As one child said, "By using the planner, people, like I did, you carry on doing it because you know that the planners there and you want to use it. Then like when its over you just continued doing it and its just to keep you fit really." A few children reported continued use of some of the resources (e.g., a couple had continued to occasionally use recipes they had got from the Space Cafe planet) and a small number reported participating in other mass participation events (e.g., a 5 km run/walk for charity). It was not clear why more had not continued to use resources or participate in other activities. The overall feeling that emerged from the focus groups was that when there were events organised for them to participate in or resources were provided they were willing and happy to work towards the event or use the resource, but this had not led to continued change when the event was over or the resource removed. Participants who had taken part in further events/activities had for the most part done this with their parents or siblings. In addition when asked about who or what helped them have healthy lifestyles family members (and most often parents) were generally mentioned first, followed by teachers and friends. The main ways parents helped included; doing sport with them, taking them to sporting activities, paying for the sporting activities, buying them sporting equipment, going on sporting holidays, encouraging them to 'go out and play' rather than watch television, and giving them healthy foods. Grandparents were also mentioned as being involved with transporting their grandchildren to sporting activities and teaching them to play different sports such as golf. However, some children also described how these people could at times hinder participation in healthy lifestyles by, for example, limiting time outside or providing unhealthy snack foods. For example one participant said: "Most of the time we're not allowed out to be able to run around and things because my Mum wants to keep us safe".

## Discussion

This follow-up evaluation of the GF2R programme showed that the positive changes in physical activity and body composition observed at the end of the intervention [16] were not sustained, and almost 2 years after the intervention there were no significant differences between the two groups for physical activity (both groups had increased physical activity) and some evidence for poorer outcomes in body composition within the intervention group. The general increase in physical activity across both groups may reflect seasonal differences in physical activity as the baseline measures were conducted in autumn/winter and the follow-up measures were conducted in spring/summer.

At post intervention both groups had increased their physical activity but the increase was significantly greater in the intervention group (e.g., 1532 steps/day greater increase in the intervention group [16]). At follow-up the difference in increase was smaller and non-significant (e.g., 678 steps/day greater increase in the intervention group). Given that the difference between groups is much lower at follow-up the non-significant result is more likely to be a true reflection of no difference between the groups rather than a result of loss of power due to diminishing sample size. There are a number of possible explanations for the lack of sustained behaviour change observed.

The intervention itself lasted for only 10 months, which may have been insufficient. Additionally the intervention may not have been intensive enough. Two primary school interventions that have demonstrated sustained intervention effects over 3 [5] and 4 [4] year follow-ups were longer interventions in the first place (3 school years [5] and 6 school years [4]) and also involved a much more intensive programme of intervention. In the current study teachers were free to use their professional judgement to choose which parts of the intervention would best work for them and their pupils, this may have resulted in low levels of exposure to different components of the intervention. Additionally, by conducting the intervention over a longer period of time participants would have been repeatedly exposed to key messages and intervention activities potentially resulting in a longer lasting effect. Even in the two studies [4,5] which continued to show a significant intervention effect for physical activity, the differences between intervention and control groups were narrowing in magnitude over time [5] suggesting that more research is needed to investigate the best way to create sustained change as children progress through to adolescence and adulthood.

Long-term interventions may be particularly important in children as the type and purpose of physical activity undertaken varies with age. At young ages basic movement patterns are developed which form the foundation for activity at later stages [21]. With growth, maturation, and experience, these basic movements are coordinated into more complex movement patterns that characterise the free play, games and sports of older children [21]. Malina [22] suggested that until approximately 8 - 10 years the main emphasis is on greater physical activity and particularly motor skills. After 8 - 10 years, the emphasis becomes increasingly focused on prescriptive physical activity, with an emphasis on health, fitness and behavioural outcomes. These changes, alongside other physical, social and cognitive changes occurring through childhood and adolescence perhaps suggest that long-term interventions that adapt to the changing needs of the young person are required to support sustained engagement with physical activity, and that it is perhaps unrealistic to expect long-term impact of a one year intervention within such a dynamic system. It is likely that the nature and content of the interventions will need to vary as children develop, and there is evidence of programme evolution in both Manios [6] and Nader [5]. van Sluijs et al. [15] suggested that traditional cognitive approaches, potentially combined with environmental approaches, may increase activity among adolescents and older children (> = 10 years), but more structural environmental or policy changes might be needed to change younger children's physical activity.

The variance estimates for the physical activity measures reported in Table 2 demonstrate that over time the variance within an individual is greater that the variance between individuals. This is most likely to be a reflection of the developmental changes discussed previously. This suggests that behaviour changes as children develop, and for example gain greater autonomy and independence, are greater within an individual than the heterogeneity between subjects at any one point in time.

As with other studies [14] the focus groups in this study highlighted the importance of parents in promoting physical activity in children. The role of parents may be particularly important in maintaining change through the provision of ongoing encouragement and tangible support for participation. Greater emphasis on engaging and supporting parents within school-based interventions may be required to facilitate long-term change.

By far the majority of teachers in this study had not continued to use the resources provided during the intervention period. While the teachers provided many potential explanations for this, an important explanation may lie in the philosophy of the intervention itself. The intervention did not mandate the use of any resource and teachers were free to choose what to use. In an environment where teachers constantly seek to meet changing curriculum demands and emphasis from regulatory authorities (see comments in focus groups) it may be that teachers do not have the time to embed successful intervention strategies long-term because they have been pulled off in a different direction. Continued support to teachers and emphasis on the outcomes of strategies may be needed to make sure that successful strategies are not overlooked in the future, or a strategy of phased support may be required. For example, Haerens et al [23,24] reported on a 2-year long school-based intervention in which the support offered to teachers reduced over time. In the first year the teachers were provided with guidance and support from the research team to help get the intervention started, but in the second year this external support was decreased with the intention of increasing the autonomy of schools. It was hypothesised that the second year would not lead to additional positive changes but it was hoped that the original changes would be sustained. Results showed significant positive intervention effects for physical activity at year 1 [23] which were sustained during year 2 [24]. While the long-term effects of this intervention have not yet been evaluated, the strategy of phased support may provide one avenue to ongoing intervention success. This would support the comments from the teachers in the current study who suggested that continued input from the intervention team would have been helpful and welcomed.

Teachers and pupils alike recognised the importance of the highlight events within the intervention but opportunities like this were not subsequently provided by the schools. This is not surprising given the many time demands and responsibilities of teachers. Ways to continue to provide highlight events without increasing the demands of teachers need to be explored. Likewise, pupils liked the holiday materials and other resources provided and reported that they thought they were useful, however, very few children had taken the idea of planning forward and continued to use some sort of action planner. Further understanding of how best to facilitate long-term use of such approaches is required.

The body composition results at follow-up are difficult to explain from the data available, particularly in light of the general increase in physical activity in both groups. Nader et al [5] also reported no significant intervention effect for body composition variables at 3-year follow-up in the CATCH trial. Singh et al [25] reported on the results of an 8-month multi-component health promotion intervention aimed at preventing excessive weight gain in young adolescents (12-14 years at baseline). Intervention effects at the end of the program and at 4-month and 12-month follow-up were presented. At the 12 month follow-up intervention effects remained for sum of skinfolds in girls. However, no intervention effects were observed in sum of skinfolds at any time point in boys, and no intervention effects were observed for BMI at any time point in both boys and girls. It is obvious that challenges remain in identifying effective strategies that result in long-term positive changes in body composition among youth. The possibility of negative rebounds when interventions are removed needs further investigation and may have important implications for the maintenance of a healthy body composition.

Although this study has several strengths (e.g., objective measures of physical activity, multiple measures of body composition) several methodological limitations should be acknowledged. Although 70% of participants were still present at follow-up the loss to follow-up was greater in the intervention schools. Overall, there was no difference (p > .05) at baseline in age, body composition, steps/day, or minutes of MVPA between those present at follow-up and those absent. Due to the local media content it was not possible to conduct a randomised control trial. However, schools were matched on key variables and there is debate as to the appropriateness of randomised control trials for evaluating health promotion interventions [26,27]. The group level matching was not reflected at the individual level resulting in the intervention group being of lower socioeconomic status than the control group. However, including socioeconomic status in the analytical models did not improve the fit of the models suggesting that these differences did not influence the outcomes.

The initial evaluation of the GF2R programme showed that the strategies employed within the intervention were effective in producing short-term changes in physical activity and body composition; however, this follow-up evaluation shows that the changes were not sustained. Thus questions remain as to how to effect long-term favourable changes in health behaviours in young people. Longer term interventions, with greater links with families are most likely required but the exact nature and contribution of this involvement remains unclear [28]. Further support to schools and teachers is also likely to be required but the best way to provide this within an already busy curriculum needs further attention.

## Competing interests

The authors declare that they have no competing interests.

## Authors' contributions

TG, participated in the design of the study, managed the data collection process, conducted the analysis and drafted the manuscript. JG, HM, SB, and MN participated in the design of the study, the data collection process and helped to draft the manuscript. AN supervised the statistical analyses. All authors read and approved the final manuscript.
